# Application of Machine Learning Techniques in the Prediction of Surface Geometry

**DOI:** 10.3390/ma19040661

**Published:** 2026-02-09

**Authors:** Aneta Gądek-Moszczak, Dominik Nowakowski, Norbert Radek

**Affiliations:** 1 Faculty of Mechanical Engineering, Cracow University of Technology, 31-155 Cracow, Poland; aneta.gadek-moszczak@pk.edu.pl (A.G.-M.); dominik.nowakowski@doktorant.pk.edu.pl (D.N.); 2 Faculty of Mechatronics and Mechanical Engineering, Kielce University of Technology, al. Tysiąclecia Państwa Polskiego 7, 25-314 Kielce, Poland

**Keywords:** machine learning, stochastic processes, recurrent neural networks, Monte Carlo method, generative adversarial networks, expectation–maximization, predictive modeling, materials science, surface geometry

## Abstract

The article presents an attempt by the authors to generate a digital representation of the analyzed surface layer of WC-Co-Al_2_O_3_ coating deposited by the ESD method. The WC-Co-Al_2_O_3_ surface layer is superhard and abrasion-resistant, significantly increasing the exploitation time of working elements. The authors aim to develop a method for generating series of digital surfaces with similar geometry parameters based on data collected through profilometric analysis. Therefore, the advanced integration of machine learning (ML) techniques with classical statistical approaches for modeling and predicting stochastic processes. While traditional models such as ARMA/ARIMA and hidden Markov models (HMMs) offer mathematical rigor, they often impose assumptions of stationarity and linearity, which limits their application to complex, noisy data. This paper proposes a model for surface geometry generation based on experimental data that combines recurrent neural networks (RNNs) and Monte Carlo simulation. Additionally, the study reviews emerging methods, including generative adversarial networks (GANs) for stochastic simulation and expectation-maximization (EM) algorithms for parameter estimation. An empirical case study on WC-Co-AL_2_O_3_ surface geometries demonstrates the effectiveness of ML–stochastic hybrids in capturing both deterministic structures and random fluctuations. The findings underscore not only the benefits but also the limitations of such models, including high computational demands and interpretability challenges, while proposing future research directions toward physics-informed ML and explainable AI.

## 1. Introduction

The properties of the surface layer, including its geometry, are among the key factors determining the exploitation properties of engineering components. Applying surface layers to enhance the properties of manufactured parts under service conditions has a long-established tradition. Surface coatings can improve resistance to environmental factors and mechanical loads, significantly extending component service life. The process of selecting an appropriate surface layer composition of materials and developing suitable manufacturing and deposition parameters is time-consuming and expensive because it is requires conducting numerous experiments, measurements and analysis.

This study focused on just one chosen aspect of the quality analysis of deposited layers, namely the surface geometry that has a strong impact on the tribology and exploitation properties. The analysis is based on the WC-Co-Al_2_O_3_ layers deposited by the Electro-Spark Deposition system (ESD method).

ESD coating system is particularly beneficial for repairs of jet engine turbines, landing gear hydraulics, and tactical vehicles. Reducing costs in the automotive industry by applying a protective coating to a wide range of automotive parts is possible thanks to the ESD process, which reaches hard-to-reach areas because it is manually controlled.

The properties of the surface layer have a strong impact on the exploitation life of parts of machines and devices. In most cases, damage to the surface layer of individual components is caused by friction and wear processes. It is therefore important to develop a new surface layer with specific properties that significantly increase the resistance to tribological wear and significantly extend the life of structural materials. In this process, AI-aided methods may play an important role in developing new surfaces and optimizing the process of the deposition. This approach can result in shorter time for the research and implementation process which will reduce the costs of experimental research and, with faster implementation, will reduce the costs of machine downtime due to damaged contact surfaces.

The primary objective of the research is to develop the model for generation of digital surfaces based on experimental dataset obtained from profilometry tests. The generation of sets of digital representation of real surface will enable the expansion and diversification of the database, which could then be used to develop a predictive model in further and more advanced stages of research.

Predictive models would be a future solution to significantly improve the production of surfaces with characteristics in line with customer requirements, eliminating the need for additional experimental testing and relying solely on data analysis [1,2,3]. Machine learning enabled the analysis of large datasets and optimization of predictive models in many areas of science and in the analysis of production processes. Researchers also used static model checking to verify compliance with specific criteria [4]. Proposed strategies achieved promising results on analyzing the spreading COVID-19 which provides valuable information that authorities could use to working out the strategy for stopping the spread of the virus [4]. Deep reinforcement learning (DRL) has been successfully applied in logistics and supply chain optimization, particularly to problems that have their source in dynamically changing and uncertain factors that significantly impact delivery operations. Application of the DRL-based method for supply chain optimization resulted in better results than traditional optimization approaches, such as distributionally robust optimization and sample average approximation. The advantage of these models lies in their flexibility, which delivers dynamic delivery routes. By learning from previous experiences, reinforcement learning systems gradually improve their performance in real-world settings [5]. Another example of the use of the combination of machine learning and stochastic methods can be found in structural engineering. Combinations of numerical analysis, machine learning, and stochastic methods was applied to predict wall resistance under seismic activity. Machine learning predictive models estimate wall behavior under external forces, eliminating the need for costly physical testing. Stochastic methods are employed to account for uncertainties associated with material properties and seismic impacts, resulting in more reliable models [6].

## 2. AI Methods in Material Science

Interest in AI methods for analyzing material structures and their impact on mechanical, chemical, and operational properties is increasing. An examination of the latest scientific reports in this field clearly shows that an increasing number of researchers are attempting to develop models capable of predicting material properties based on data describing their chemical composition and structural components [7,8,9,10,11].

By utilizing large experimental datasets, the developed models enable the prediction of mechanical properties [12,13], which significantly reduces the time and cost associated with introducing new materials into production. Diwakar et al. (2024) [14] proposed using machine learning techniques for surface coatings of the FeCoCrNiMo alloy on EN24 alloy steel. The authors employed three models: a Support Vector Machine (SVM) with a radial basis kernel, the K-Nearest Neighbors (KNN) method, and a Multilayer Perceptron (MLP) [14]. These models were applied to predict and assess the potential for metallurgical bonding formation and bead morphology in the laser cladding process, as well as to optimize the profile of a multilayer surface coating. Zimmermann et al. (2024) proposed applying a standard neural network model consisting of an input layer, two dense layers with 512 neurons each, and a single output neuron [15]. The Rectified Linear Unit (ReLU) function was used as the activation function, while the output layer employed a linear activation function. In this model for the training stage, the ADAM (Adaptive Moment Estimation) optimizer was applied, and the mean squared error (MSE) was used as the loss function. The prediction results achieved very good R^2^ values and low error metrics, which confirmed the proposed method’s efficiency.

Introducing a deep learning method in an advanced approach to intelligent metamaterials [16], which are not only a static structure but a complete metasystem, which integrates four fundamental functions within a single structure: sensing, actuation, control, and communication. This visionary approach to designing metamaterials, which accounts for the high-level integration of electronic and active materials, enables the creation of feedback loops. Feedback loops allow the material to “feel” deformation and instantly change its stiffness to compensate. Precise modeling of this kind of structure, due to its nonlinearity and geometric complexity, can be analyzed and controlled using algorithmic models supported by surface RNNs and point cloud processing methods, whose utility for the digital surface generation method is analyzed by the authors in this paper.

Stochastic processes, which the authors propose as one of the elements in a designed AI predictive model, are mathematical models used to study random phenomena.

In materials science, methods combining deep machine learning with stochastic finite element methods have been used to predict the strength of composites without destructive testing. This hybrid method integrates physics-based modeling, ML, and stochastic analysis to predict material performance under full load. The Monte Carlo method, coupled with deep learning, further refines predictions by simulating discrepancies between numerical and experimental data, thereby providing reliable uncertainty quantification [16]. Finally, in the study of complex dynamic systems, researchers have introduced algorithms such as FODS-NAR, specifically designed for stochastic systems of fractional order. These systems, characterized by fractional calculus and memory elements, are difficult to analyze with traditional approaches due to their complexity. The FODS-NAR algorithm, based on deep learning, demonstrated excellent predictive accuracy, particularly in managing random and fractional components of such models. Its unique strength lies in its ability to capture dynamic behaviors with reduced error and improved reliability, highlighting the utility of combining machine learning with stochastic modeling for highly complex systems [17].

These examples demonstrate that the combination of machine learning and stochastic process theory not only improves predictive performance but also extends the range of problems that can be addressed under uncertainty. The integration of data-driven algorithms with probabilistic modeling thus represents a promising direction for advancing both theoretical understanding and practical applications of stochastic systems.

## 3. Materials and Methods

### 3.1. Materials

The analysis focused on analysis of a coating deposited on the substrate material C45, by Electro-Spark Deposition (ESD) WC-Co-Al_2_O_3_ layers (84% WC, 6% Co, 10% Al_2_O_3_). The coatings were fabricated at the Centre for Laser Metal Technologies in Kielce [18].

WC-Co-Al_2_O_3_ coatings were produced using the Electro-Spark Deposition (ESD) process and applied as tribological layers. The use of nanostructured electrodes made of WC-Co-Al_2_O_3_ cemented carbides enabled the deposition of coatings with increased density, thickness, hardness, and wear resistance. The ESD process allows the formation of hard, wear-resistant coatings with high adhesion to various substrate materials, such as steel, nickel, aluminum alloys, and ceramics. In the coatings investigated, tungsten carbide (WC) is the dominant phase.

Another advantage of the ESD technology is the low heat input during deposition, which allows its application in the repair and restoration of components susceptible to heat-affected zone cracking, as well as in the regeneration of mechanical parts to extend their effective service life.

The WC-Co-Al_2_O_3_ coatings were deposited using the EIL-8A device, manufactured by TRIZ in Sumy, Ukraine with manual electrode feed. The following deposition parameters were used:

- voltage: U = 230 V,
- capacitance of capacitors: C = 300 µF,
- current intensity: I = 2.2 A,
- exposure time: τ = 2 min/cm^2^.

**Figure 1:**
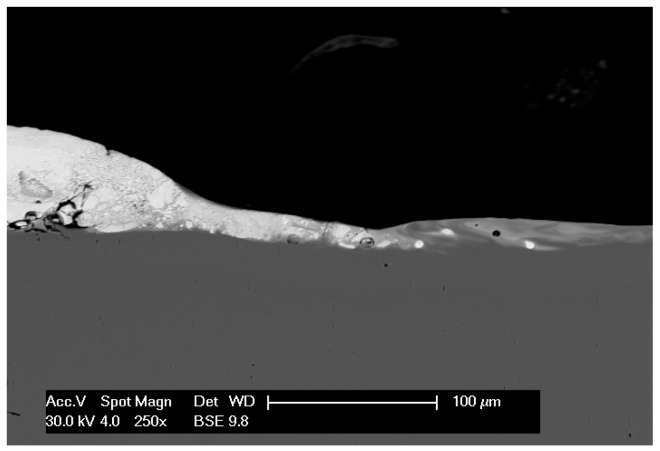
Cross-section of the sample with WC-Co-Al_2_O_3_ coating, magnification: 250×.

The microstructure of the cermet coatings, both before and after laser treatment, was examined using a high-resolution Quanta 3D FEG scanning electron microscope produced by FEI Company in Brno, Czech Republic (SEM/FIB). Visual assessment of deposited layer can by reinforced by automatic image analysis, to quantify the layer defects like local discontinuity, inner cracks, consistency with the base material or thickness of the layer. The outcome is a set of parameters which allows us to assess the level of homogeneity and quality of deposited layer. As can be observed in Figure 1, SEM images reveal the small crack in the layer, the diversity of the thickness, and good coherence of the layer with the base material.

Analysis of the microstructure on SEM provide the information from the limited area—the cross-section of prepared samples. In order to have more complete information about the deposited coating, the 3D analysis were performed. To carry out this evaluation, three-dimensional datasets were obtained at the Micro and Nano X-ray Tomography Laboratory of AGH University of Science and Technology (Poland) using the Nanotom 180N system from GE Sensing & Inspection Technologies Phoenix|X-ray GmbH, manufactured in Wunstorf, Germany [19]. A tungsten target served as the X-ray source, and the polychromatic radiation was filtered with a 1 mm copper filter. The X-ray tube parameters were I = 170 µA and V = 130 kV. A total of 2100 projections were collected, each averaged over 10 integrations, resulting in an overall scanning time of approximately 400 min. The reconstructed volume achieved a resolution of 1 µm, and the scanned region of the specimen measured 2.2 mm × 3.1 mm × 3.0 mm.

**Figure 2:**
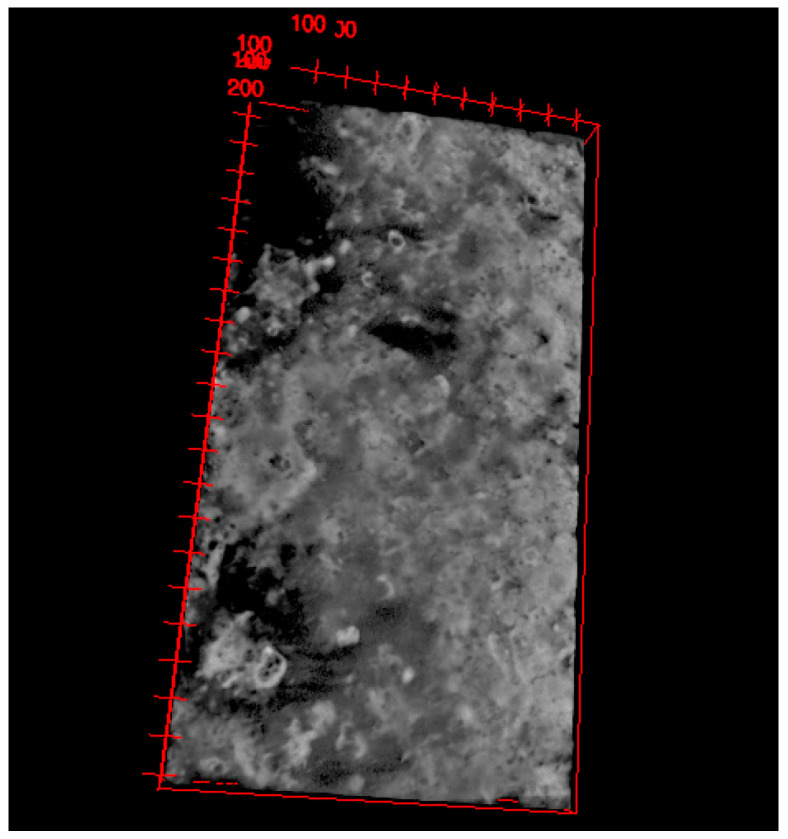
Three-dimensional reconstruction of WC-CoAl_2_O_3_ layer.

The obtained set of cross-section images was used to generate the reconstruction of three-dimensional visualization of the tested sample (Figure 2) in ImageJ 2 2.17.0 open software [20]. Due to significant difference in density of the material of the base and the coatings, on the image, the higher values of the pixels in the cross-sections represents the coating and lower values, due to lower X-ray absorption, represent the material of base. The reconstruction revealed the diversity in the thickness of the coating and the complex, irregular surface geometry. Mean thickness of the coating is 36.6 microns and the standard variation is 29 microns. Measurements confirmed the irregular geometrical structure of the coating.

The most precise data describing the geometry of analyzed surface, is profilometry.

**Figure 3:**
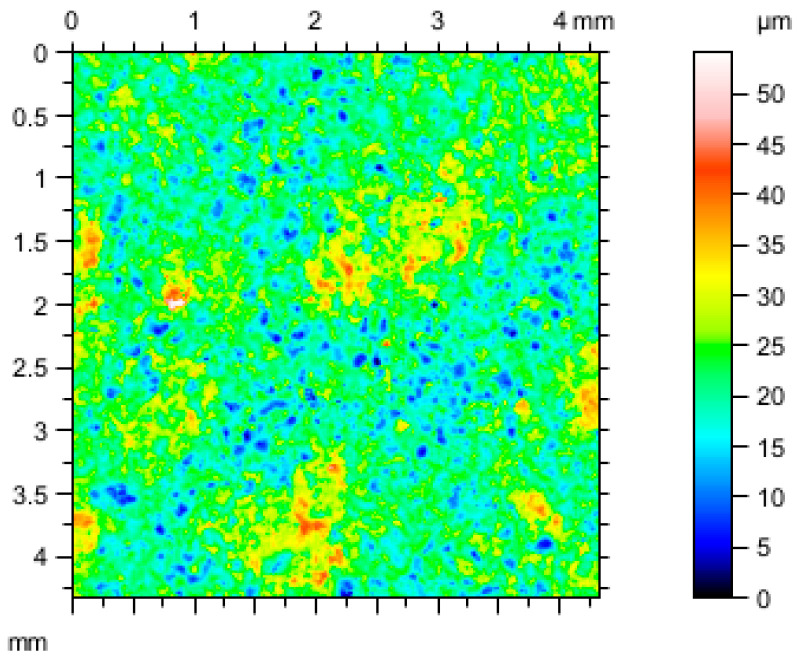
WC-Co-Al_2_O_3_ surface layer—results from profilometry presented as 2D color map.

The tests were performed using a Talysurf CCI optical profilometer, manufactured by Taylor Hobson Ltd., Leicester, UK, which utilizes Taylor Hobson’s patented coherence correlation algorithm, enabling measurements with a z-axis resolution of less than 0.8 nm. The measurement results are recorded in a 1024 × 1024 matrix of measurement points, which, with a 10× lens, provides a measured area of 1.65 mm × 1.65 mm and a horizontal resolution of 1.65 μm × 1.65 μm [21]. Three-dimensional surfaces and their analysis using TalyMap Platinum 7.4 software allowed for precise description of the geometric structure of the examined surfaces (Figure 3). The geometrical parameters, according to ISO 25178 international standards for geometric product specification, and surface texture were calculated (Table 1).

Three-dimensional surface structure, according to the standard, is characterized by a set of parameters which includes: Sq [µm]—root mean square heigh deviation for the mean plane. It describes the overall roughness of the surface and is particularly sensitive for high peaks and deep valleys; Sa [µm]—arithmetical mean high, calculate the mean arithmetical absolute value of deviation for the mean plane; parameters characterizing the histogram of the high; and Ssk—measure the asymmetry of height distribution and Sku describe the sharpness of the height distribution. Sp [µm] is the value of the maximum peak height, Sv [µm] refers to maximum valley depth and Sz [µm] is a maximum height and is calculated as sum of maximum peak and maximum valley depth. The results of the geometrical analysis calculated in TalyMap Platinum software are presented in Table 1.

These sets of parameters provided are complementary information about the examined layer with a textural description, which influences its functional and optical properties. An additional advantage of profilometric testing is the complete reproduction of the surface with higher accuracy than possible with microtomographic testing, due to its higher measurement precision. Therefore, when considering a data source that could be used to test the concept of creating a digital surface model using machine learning methods, the use of data from profilometric testing were the most accurate. This data, in the form of a dataset, in which each measurement point is characterized by three values: an X and Y coordinate corresponding to the measurement point, and a Z coordinate, the value of which corresponds to height of the surface in the reference point.

### 3.2. Algorithm Description

The use of machine learning methods in materials science and related fields demonstrates their wide range of possible applications [22,23,24]. The introduction of neural networks into machine learning models has enabled data exploration on a previously unprecedented scale.

Considering the choice of appropriate approaches to process point clouds data, three types of architecture are in the field of interest: PointNet, CNN and RNN. The PointNet architecture allows us to process 3 dimensional point cloud directly without the need of conversion to the grid (voxels) or 2D images [25]. PointNet model is a proper model for unordered data, changing the order of points in a file, which does alter the object itself, and what leads to ignoring the local structures. In considering the type of surface, where the technique of deposition or any surface treatment (e.g., laser processing) leads to the creation of a certain texture, this feature of the model disqualifies its use according to our considerations.

CNN models need structuralized data, for example, transformation to a grid or voxels or 2-dimensional. CNN models are used for the detection of specific image features like edges, corners, or texture. In this model, convolutional filters slide over the data which reduces the number of parameters compared to a fully connected network. Thanks to pooling layer CNN can build hierarchical representations from simple edges to complex object parts. This technology is very well developed; algorithms like ResNet or EfficienNet are stable and are supported by PyTorch 2.8.0 or Tensorflow 2.19.0. But it needs to be remember that converting point into voxels always involves rounding. If the voxel are large, fine details are lost, and if they are small, memory usage grows very quickly, with N^3^. Standard 3D convolutions are very expensive for GPUs compared to working directly with point coordinates [26].

The proposed model is based on recurrent neural networks (RNNs), which are deep learning methods. The choice of RNNs was motivated primarily by the type of data being analyzed, which can be defined as sequential surface data, and this is precisely the type of data specified for RNNs [27,28,29]. Recurrent neural networks, a class of deep learning algorithms designed explicitly for sequential data, are particularly well-suited to this task. By training on historical sequences, RNNs can model time-dependent stochastic behavior and generate accurate predictions. Another key application is parameter estimation. In practice, the actual underlying process that generates the data is often unknown. Traditional estimation methods, such as maximum likelihood estimation, can be computationally demanding and prone to inaccuracies in complex systems [29]. Machine learning approaches, such as the expectation–maximization (EM) algorithm, provide more flexible and efficient tools for estimating parameters by iteratively inferring latent variables and refining model parameters. By using algorithms such as EM, the parameters of complex stochastic processes can be estimated with higher accuracy.

Random walk is a stochastic process where the next step taken is determined by a probability distribution, typically a Gaussian distribution. In recent years, machine learning algorithms have been used to model and analyze random walks, providing new insights and improving the accuracy of predictions. As proposed by authors, model machine learning is used to model and analyze random walks. One of the most important applications of random walk models is prediction. Traditional methods for predicting random walks involve statistical models such as autoregressive models or Markov chains [30,31]. However, these models often make assumptions about the underlying process that may not be accurate. Machine learning algorithms, on the other hand, can learn the underlying patterns in the data without making assumptions about the process. An example of this type of algorithm is the recurrent neural network (RNN), which can be used to model sequential data such as random walks. By training an RNN on a sequence of observations from a random walk, the algorithm learns to predict the next step based on past observations.

Another application of machine learning in random walk modeling is parameter estimation. In many cases, the underlying distribution of a random walk is not known, and it is necessary to estimate the parameters of a statistical model that best fits the data. Traditional methods for parameter estimation, such as maximum likelihood estimation, can be computationally expensive and may not always provide accurate estimates.

Machine learning algorithms such as the expectation-maximization (EM) algorithm can be used to estimate the parameters of a random walk more efficiently and accurately. The EM algorithm works by iteratively estimating the distribution of unobserved variables in a statistical model and then updating the parameters of the model based on this distribution. By using machine learning algorithms such as the EM algorithm, researchers can estimate the parameters of complex random walks more efficiently and accurately. A third application of machine learning in random walk modeling is simulation. Simulation is an important tool for understanding the behavior of random walks and evaluating the performance of statistical models.

Machine learning algorithms such as generative adversarial networks (GANs) can be used to simulate the behavior of a random walk more efficiently and accurately [31,32]. GANs are a type of deep learning algorithm that can learn to generate realistic samples from a given distribution. By training a GAN on a set of observations from a random walk, the algorithm can learn to simulate new samples from the same process.

Accurate prediction of surface coordinates can have significant applications, ranging from virtual object rendering to robotic path planning. In the paper, we present the use of recurrent neural networks in combination with the Monte Carlo method for surface coordinate prediction.

Summarizing up RNNs is used for modeling sequences and for capturing the temporal dependencies present in surfaces’ behaviors. The Monte Carlo method is used to estimate the probability distributions of uncertain variables. By combining these two techniques, developed model is a predictive model that can capture the stochastic behavior of surfaces and provide probabilistic predictions.

**Figure 4:**
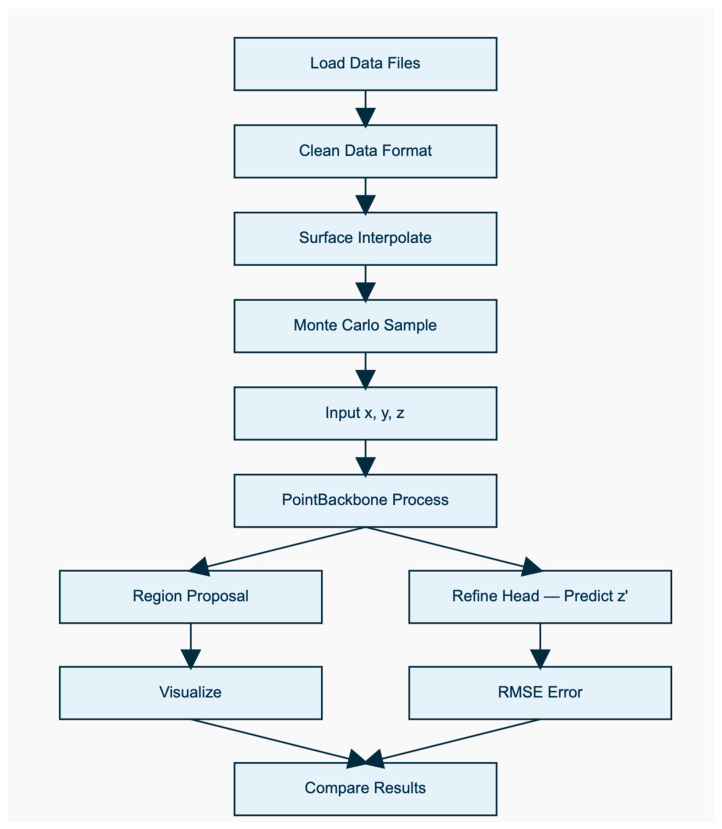
Diagram of surface RCNN model workflow.

The main stages of proposed methodology, which can be applied to generation the set of digital representation of analyzed surface consist of 6 stages:

1. Data Preparation: Collect and preprocess the historical data of the surface coordinates. This data should include a sequence of surface coordinate observations over time, along with any relevant contextual information, such as surface properties, lighting conditions, and environmental factors. It is important to ensure that the data is representative of the stochastic behavior of the surfaces to obtain accurate predictions.
2. RNN Training: Train an RNN model using the prepared data. The RNN should be configured to capture the temporal dependencies in the data, and can be designed as a sequence-to-sequence or sequence-to-one model, depending on the specific problem. The RNN should take the past surface coordinates as input and predict the next surface coordinates as output. Diagram of surface RNN model workflow was presented on Figure 4.
3. Monte Carlo Simulation: Use the trained RNN model to generate multiple future trajectories of surface coordinates. Starting from an initial surface coordinate, input it into the RNN to generate the next surface coordinate, and repeat this process iteratively to generate a sequence of future surface coordinates. Repeat this process multiple times to obtain a set of Monte Carlo simulated trajectories.
4. Statistical Analysis: Analyze the generated Monte Carlo trajectories to estimate the probability distribution of the surface coordinates. This can be done using statistical techniques, such as histogram analysis, kernel density estimation, or other relevant methods. The estimated probability distribution can provide insights into the uncertainty associated with the surface coordinate predictions.
5. Prediction and Visualization: Utilize the estimated probability distribution of surface coordinates to make predictions and generate visualizations. For example, you can use the predicted probability distribution to generate heatmaps or contour plots to visualize the uncertainty in the surface coordinates. You can also use the predicted distribution to estimate the likelihood of certain surface coordinate values or events occurring, which can aid in decision-making.
6. Evaluation and Refinement: Evaluate the performance of the combined RNN-Monte Carlo algorithm by comparing its predictions with actual surface coordinates. Refine the algorithm as needed based on the evaluation results, such as adjusting RNN model hyperparameters, improving data quality, or incorporating additional contextual information.

The proposed model was implemented in Python with PyTorch library. The neural network was prepared and trained in the Google Colab environment using Nvidia Tesla T4 GPU manufactured by TSMC in Taiwan, acceleration. Running on Ubuntu 22.04.4 LTS with Python 3.12.12 interpreter. Libraries such as numpy 2.0.2 and PyTorch 2.8.0 were used for the training process. The proposed model was implemented in Python with PyTorch library. The neural network was prepared and trained in the Google Colab environment using Nvidia Tesla T4 GPU acceleration. Running on Ubuntu 22.04.4 LTS with Python 3.12.12 interpreter. Libraries such as numpy 2.0.2 and PyTorch 2.8.0 were used for the training process. NumPy is the most important Python tool for working with numbers and large datasets. It allows users to store data in multi-layered tables (arrays) and perform fast calculations—like sorting, math, and statistics—much more quickly than standard Python would allow [4]. As an open-source framework, PyTorch optimizes the machine learning workflow between initial research models and robust production environment. Built to offer maximum flexibility and speed, PyTorch supports dynamic computation graphs, enabling researchers and developers to iterate quickly and intuitively. Due to its Pythonic architecture and integration with native Python utilities, the framework provides environment for the large-scale development and optimization of deep learning architectures [27].

Authors have chosen the LSTM-based RNN model due to the specific nature of the surface modeling along the X axis. In the code, points are sorted by X-coordinate (torch.argsort(pointsdf, 0)), which transforms the point cloud into a one-dimensional sequence. The surface is treated as a sequential process along the X direction, similar to how the coating deposition moves linearly along this axis. The LSTM tracks the entire path of the points, remembering where previous points were located to better understand the overall shape.

Bidirectionality enables the model to consider both past and future points in the sequence X, which is crucial for accurate surface reconstruction.

RNN/LSTM is therefore a natural choice for this data topology, where the directionality of X defines the sequential structure of the surface.

The applied model architecture uses a hierarchical structure in which data are processed in stages, gradually increasing and refining the features of each point (Table 2).

LSTM#1 is the first stage of data processing, based on feature extraction. The model processes the point sequence in both front-to-back and back-to-front, allowing it to gather context for each point from all other points in the sequence. The network expands the 3-dimensional input into a 128-dimensional hidden representation per direction, which results in a 256-dimensional feature vector after concatenation.

LSTM#2 is a layer that refines features. It takes the 256-dimensional features from LSTM#1 and processes them with another two-layer bidirectional LSTM. The final output dimension is 512, providing a rich representation for each point. A dropout rate of 0.1 is applied to both LSTMs to reduce overfitting and improve generalization to unseen point cloud shapes.

LayerNorm (Table 2) implements the final stage of analysis, preparing the data for the downstream task. It stabilizes training by normalizing the 512-dimensional features. The data is reshaped from (B, N, 512) to (B, 512, N), which is the standard format for Point-Net-like architectures that use 1D convolution or global pooling along the point dimension.

**Figure 5:**
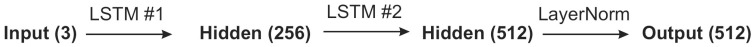
The scheme of data transformation in applied model.

The transformation of a single point’s representation via the backbone follows the path shown in Figure 5.

The proposed scheme is effective if we assume the sequential nature of surface deposition and capture global dependencies through the recurrence mechanism rather than just local spatial grouping.

The model was trained for 100 epochs using an Nvidia Tesla T4 GPU with Turing architecture, which is equipped with 40 SM units and 16 GB of GDDR6 memory (15 GB available to the user). The card’s memory bandwidth is approximately 320 GB/s, and its performance reaches 8.1 TFLOPS in single precision (FP32) and up to 65 TFLOPS in mixed precision mode (FP16) using Tensor cores.

Training was performed using FP16 acceleration to speed up calculations and optimize memory usage.

Proposed algorithm executes the follow steps:

1. Load Data File/Clean Data Format—loading and changing the data format of 3D points (x, y, z coordinates) from the profilometer located in .txt files and creating a set of surface points before and after processing.
2. Surface Interpolate—interpolating Z-axis values after processing relative to the common X-value range of the surface to obtain similar points on both surfaces.
3. Monte Carlo Sample—random sampling of point values before processing and introducing slight random noise to create a training dataset (Input x, y, z)
4. PointBackBone Process—processing points through a PointBackBone network consisting of a 4-layer convolutional network to extract features for each point.
5. Region Proposal—generates a prediction of point displacement in 3 dimensions based on the extracted features. This stage takes the feature tensor from the PointRNNBackbone (B × 512 × N). It passes these features through a fully connected network (512→256→128→3) with ReLU activations between layers. The result is a Δz tensor (B × 3 × N). The obtained results are treated as a “raw proposal” for local height variation, which will be refined in the next step.
6. Refine Head—combines PointBackbone and RegionProposal features along the feature axis and then passes through the layers to produce correction values for Z. The Refine Head combines the features from the backbone (512 channels) with the output from the Region Proposal (3 channels). That creates a 515-dimensional vector for each point. To keep the sequence structure, the points are sorted by their X-coordinate. Finally, this vector passes through three linear layers (515→256→64→1) with ReLU. The output is the final, one-dimensional height prediction (Z) for every point on the surface.
7. Model training—optimizer ADAM, Huber loss function, number of epochs: 50.

The choice of loss function was based on the specific nature of the data being processed. MSE Loss measures the average of the squares of the errors. It is sensitive to outliers because squaring large errors can make them dominate the overall loss, slowing down model learning on noisy data. L1Loss (Mean Absolute Error or MAE) is a way to measure the distance between a model’s prediction and the actual truth. L1Loss is more resistant to outlier points because it does not square errors, but it responds less strongly to large deviations and learns more slowly with small errors. Huber Loss combines the best of both above methods. This loss function combines the advantages of MSE and L1 Loss: it is fast and accurate with small errors like MSE, and stable with large errors like L1 Loss.

Huber Loss uses a threshold hyperparameter, Delta δ, if the error is small, within the range of δ the function acts like MSE, and if the error is outside the range, δ it acts like L1 Loss. Considering those three options, the most appropriate loss function for the analyzed data, Huber Loss, allows the model to ignore measurement noise while remaining precise on the actual object [16,17,33,34].

The model was validated on data from the same source as the training set, without using an external set or cross-validation. This approach was sufficient for testing the methods used, given the use of Monte Carlo sampling to generate a variety of point samples and the experimental nature of the current stage. Authors will test the network on new data obtained independently. The training and validation datasets will differ, ensuring a complete evaluation of the model’s generalization.

## 4. Results

**Figure 6:**
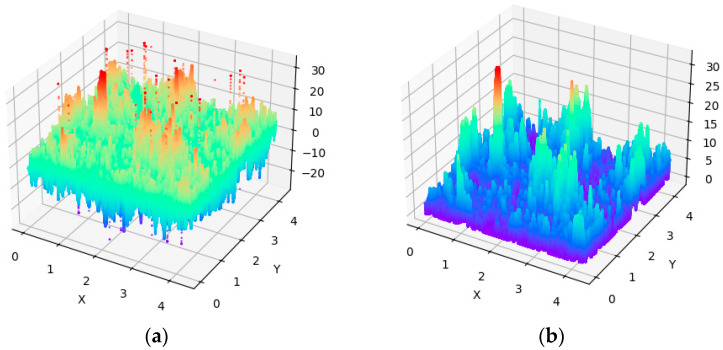
Visualization of raw data from raw file obtained from profilometry. (**a**) Visualization of raw data; (**b**) visualization of normalized data.

The results of measuring the sample with the surface of WC-Co-Al_2_O_3_. The initial phase of the conducted analyses comprised a preliminary visualization of the raw data, implemented as a three-dimensional plot represented in the form of a color map (Figure 6a).

Since the analyzed dataset exhibits substantial variability and, at the same time, visible local fluctuations in value, i.e., noise; Gaussian smoothing with a 3 × 3 kernel was applied, followed by data normalization to ensure that the minimum recorded value was set to zero. In this manner, a surface representing by 1,048,576 measurement points [x, y value] was obtained (Figure 6b).

Next the experiment was conducted due to the obtained set of digital surfaces which will be generated based on the data from profilometry. Subsequently, four surface simulations were generated that were intended to replicate the investigated WC-Co-Al_2_O_3_ layer’s surface, yet not be identical to it. The objective of this procedure was to assess whether our model can generate digital representations of the real material’s surface based on a small sample. This ability of the model to generate more data from a small sample is very important for the design of predictive algorithms, whose performance strongly depends on the amount of available input data in the case where access to the real data is limited or very expansive.

**Figure 7:**
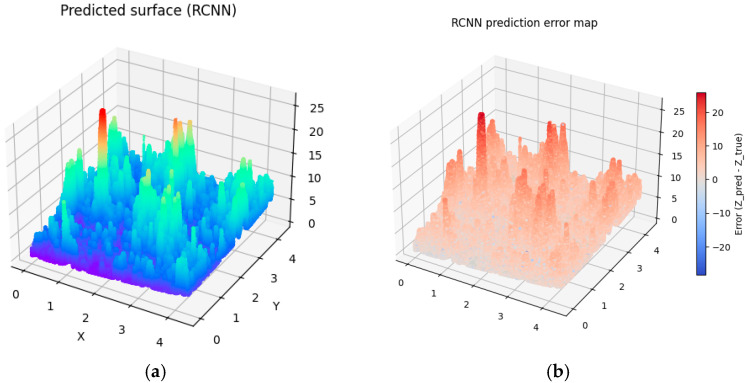
Visualization of predicted surface 1 (**a**) and prediction error map applying prediction method 1 (**b**).

**Figure 8:**
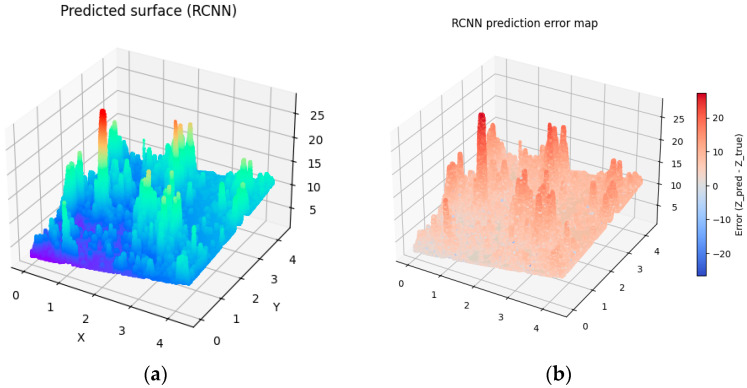
Visualization of predicted surface 2 (**a**) and prediction error map applying prediction method 2 (**b**).

**Figure 9:**
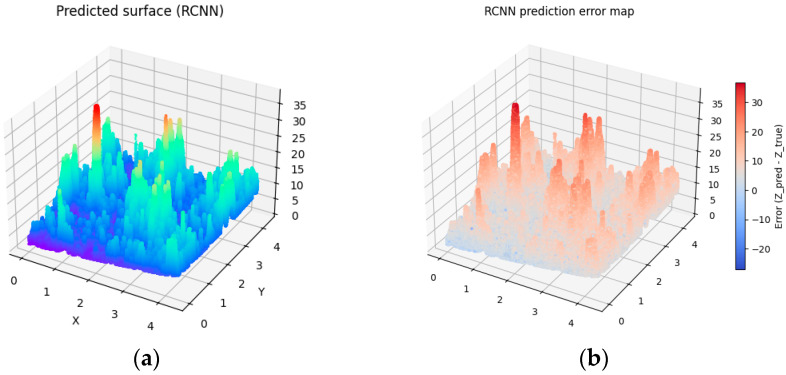
Visualization of predicted surface 3 and prediction error map applying prediction method 3 (**a**) and the prediction error map (**b**).

**Figure 10:**
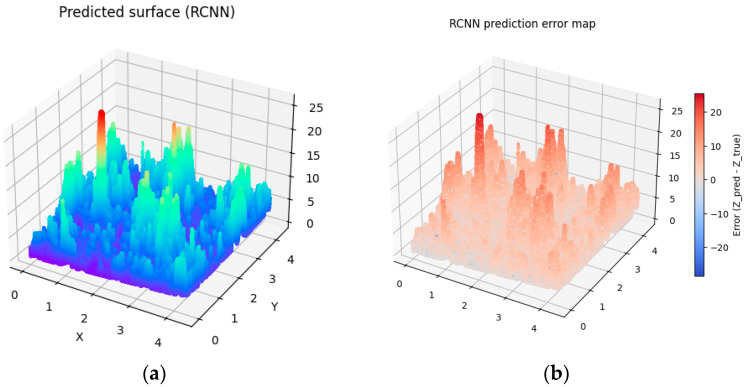
Visualization of predicted surface 4 and prediction error map applying prediction method 4 (**a**) and prediction error map (**b**).

To introduce variability into the digitally generated surfaces, differences were implemented in the number of interpolated surface points as well as in the number of random points used in the Monte Carlo method (see Table 3). As a result, four distinct generated surfaces were obtained (see Figure 7, Figure 8, Figure 9 and Figure 10).

To assess the degree of similarity between the digital-twin surfaces and the experimentally measured surface, the RMSE prediction error was calculated. The Root Mean Squared Error (RMSE) is one of the most used error metrics, indicating the degree to which the model-generated data deviate from the actual measurements. An analysis of the calculated RMSE values indicates that Surface 4, generated based on 100,000 points in the interpolated variant as well as using 100,000 points in the Monte Carlo method, achieved the lowest value, which signifies the highest level of agreement with the actual surface. However, the low RMSE values for the remaining surfaces allow us to conclude that all four simulated surfaces exhibit a high degree of similarity to the investigated surface and can be considered acceptable, which confirms the effectiveness of the proposed model.

Analyzing the obtained charts presenting generated digital surfaces, they present small differences between each other. The variety introduced by different parameters in Monte Carlo methods does not significantly impact the general shape of the surface. The results are expected because the aim of the study was to generate the family of surfaces which are not identical but similar to the other samples which were prepared in the exact same technological parameters. They present the same high diversity of peaks, height and depth of valley, and irregularity of texture.

The discrepancies in RMSE values presented in Table 3 are associated with the sampling density and interpolation quality during the generation of reference surfaces. Surface 4 utilizes the highest number of interpolation points (e.g., 10k+), providing a smooth and precise ground truth for model training. This elevated point density minimizes approximation errors. The higher number of points reduces interpolation artifacts, such as oscillations between data points, thereby enhancing prediction accuracy. A larger number of Monte Carlo (MC) samples (e.g., 50k+) in Surface 4 results in more effectively captured stochastic surface fluctuations. This reduces RMSE variance by providing a more accurate simulation of deposition processes.

Ultimately, the combination of these two parameters in Surface 4 yields the most representative reference surface, resulting in the model’s superior performance.

Based on profilometry measurements of the real sample of deposited WC-Co-Al_2_O_3_ layer, analysis of the geometrical parameters of the surface was carried out in accordance with the ISO 25178 standard and presented in Table 1 (see Section 3.1). This data was used to assess the degree of the similarity the real surface and the surface generated in the experiment. The analysis included the following key parameters: Sa (arithmetical mean hight), Sq (root mean square heigh), Ssk (skewness), Sku (kurtosis of height distribution), Sp (maximum peak height), Sv (maximum peak depth), and Sz (maximum height). The comparison of those parameters for all surfaces is presented in Table 4.

Analyzing the obtained measurement results of the geometric features for both the real surface and the generated ones, we can conclude that their values are similar, with differences within 15 percent relative. This result is consistent with the assumptions of the developed model and its purpose, namely, to generate a set of digital representations of the material surface that simulate samples produced from the same material under identical technological parameters. The only parameter that exhibits significantly greater variability in the generated surfaces than in the actual surface is Sku: in the actual sample, it is 5.163, while in the generated surfaces, it ranges from 6.670 to 13.585. The value of Sku for digital surfaces indicates that the generated surfaces have significantly sharper peaks, which may be due to an excessive concentration of values around the mean combined with the presence of extreme points. This improvement in the applied model should eliminate this phenomenon. A solution could be to use the KNN point grouping method, which should smooth the resulting surface. Another solution could be to introduce a smoothing layer, or Average Pooling, which will shift extreme values toward the mean, causing the resulting distribution to be flatter and better reflecting the characteristics of the actual surface.

## 5. Discussion

The designed model enabled the generation of four digital representations of surfaces with geometric characteristics similar to those of the surface layer under investigation, specifically the electro–discharge–deposited WC-Co-Al_2_O_3_ coating. The visualization of the generated surfaces, as well as the RMSE values and the geometric surface parameters compliant with the standard (insert standard), indicate that these representations can be considered valid analogs of WC-Co-Al_2_O_3_ surface layers, assuming they were produced under identical technological conditions.

The generation of digital surfaces corresponding to real surfaces is the first step toward developing a broader model that incorporates machine learning methods, image analysis, and deep learning. The primary research goal of the authors is to create a model capable of predicting the geometric structure of a material’s surface as a function of the manufacturing process parameters.

However, to achieve the intended goal, it is necessary to create a database containing profilometric data of surface layers produced under various process parameters. For the dataset to be suitable for developing a predictive algorithm, a very large number of samples must be produced for each variation of the process settings, followed by profilometric measurements. Such a process of preparing a training dataset is highly time-consuming and costly, and therefore difficult to implement. For this reason, the authors decided to adopt a data generation approach, analogous to data augmentation, so that the model can learn from digitally generated surfaces that closely resemble real ones.

In conclusion, machine learning is a powerful tool for modeling and analyzing random walks. By using machine learning algorithms to predict, estimate, and simulate random walks, researchers can gain a deeper understanding of complex phenomena and make more accurate predictions and decisions.

## 6. Conclusions

The results demonstrate that combining machine learning with stochastic methods has significant potential for solving predictive problems in many fields. The use of neural networks (RNNs) in conjunction with Monte Carlo simulations enables the efficient modeling of sequences while simultaneously estimating uncertainty. GANs further expand these capabilities, generating valid synthetic data.

Implementing a hybrid machine learning model to analyze real-world profilometry datasets enables the generation of digital twins of the analyzed surface, as well as a set of surfaces that simulate the surfaces on which the deposition process was performed in the same environment and parameters, without requiring any additional experimental data.

The presented model represents the first stage of the authors’ research, which aims to develop a predictive algorithm for generating a digital surface whose geometry will depend on the adjustment of the technological process. This model will enable the optimization of the layer deposition process according to the requirements of its final geometric properties.

In the planned research, the authors intend to test model modifications focused on K-Nearest Neighbors (KNN) data grouping and the implementation of a hierarchical processing architecture (set abstraction levels). This approach is expected to reduce the risk of overfitting to local outliers, as the network learns averaged local features before progressing to global features.

Machine learning is a promising tool for modeling and analyzing stochastic processes. It may be a valuable tool for analysis and prediction in materials science, from the design stage of new materials through the optimization of the production process to the prediction of the exploitation cycle. As machine learning algorithms advance, they will likely play an increasingly important role in modeling and analyzing stochastic processes, enabling researchers to solve even more complex problems. Furthermore, machine learning algorithms require large datasets, and presenting the phenomenon under study as fully as possible, for training. Interpreting machine learning algorithms can be challenging because they often make predictions based on complex and ambiguous relationships between inputs and outputs. Deep learning models often operate as black boxes, which raises some concerns about their application in areas such as medical or financial data analysis and, rather, will not be applied until its decision-making process is fully explainable. These models also require significant computing power, often specialized hardware, and careful optimization.

## Figures and Tables

**Table 1:** Parameters of the geometric structure of the coating surface WC-Co-Al_2_O_3_.

Geometrical Parameters	Value
Sq [µm]	2.302
Sa [µm]	1.826
Ssk	1.153
Sku	5.163
Sp [µm]	26.432
Sv [µm]	3.013
Sz [µm]	29.445

**Table 2:** PointRNNBackbone of proposed model.

Component	Input Dim	Output Dim	Key Parameters
LSTM #1	3 (x, y, z)	256	2 layers, Bidirectional, 0.1 Dropout
LSTM #2	256	512	2 layers, Bidirectional, 0.1. Dropout
LayerNorm	512	512	Normalizes across the feature dimension

**Table 3:** The value of the parameters in the Monte Carlo for four generated surfaces.

	Surface 1	Surface 2	Surface 3	Surface 4
Number of points in interpolate surface	100	10,000	1000	100,000
Number of points in Monte Carlo method	100	10,000	100,000	100,000
Function of loss	Huber Loss	Huber Loss	Huber Loss	Huber Loss
RMSE prediction error	3.935309	4.092348	5.756701	2.594651

**Table 4:** Comparison of the values of geometric parameters of the actual surface and the surfaces generated in the conducted experiment.

	Real Sample	Surface 1	Surface 2	Surface 3	Surface 4
Sq [µm]	2.302	2.630	2.130	2.969	2.060
Sa [µm]	1.826	1.421	1.621	2.045	1.390
Ssk	1.153	0.898	0.908	2.113	2.585
Sku	5.163	7.670	6.670	10.932	13.585
Sp [µm]	26.432	21.436	20.256	30.569	22.508
Sv [µm]	3.013	4.934	4.543	4.439	2.418
Sz [µm]	29.445	23.653	24.789	35.008	24.926
RSME error		3.935309	4.092348	5.756701	2.594651

## Data Availability

The original contributions presented in this study are included in the article. Further inquiries can be directed to the corresponding author.
